# Faster and More Robust CK Reaction Rate Estimation at 3T Using Acquisition‐Weighted ^31^P Cardiac 1D‐MRSI With Compartment‐Based Reconstruction

**DOI:** 10.1002/mrm.70408

**Published:** 2026-04-26

**Authors:** Aaron Axford, Iveta Pajanová, William D. Watson, Andrew C. Tyler, Roshan Xavier, Ferenc E. Mózes, Oliver J. Rider, Damian J. Tyler, Ladislav Valkovič

**Affiliations:** ^1^ Oxford Centre for Clinical MR Research (OCMR), RDM Cardiovascular Medicine University of Oxford Oxford UK; ^2^ Department of Imaging Methods Institute of Measurement Science, Slovak Academy of Sciences Bratislava Slovakia; ^3^ Institute of Electrical Engineering, Faculty of Electrical Engineering and Information Technology, Slovak University of Technology Bratislava Slovakia; ^4^ Department of Cardiovascular Medicine University of Cambridge Cambridge UK; ^5^ Division of Psychology Communication and Human Neuroscience, School of Health Sciences, Faculty of Biology, Medicine and Health, University of Manchester Manchester UK; ^6^ Department of Physiology, Anatomy & Genetics University of Oxford Oxford UK

## Abstract

**Purpose:**

Quantification of the creatine kinase (CK) forward reaction rate (*k*_f_) in the human heart using phosphorus magnetic resonance spectroscopy is clinically important; however, it is limited by long acquisition times, operator subjectivity in analysis, and potential skeletal muscle contamination. This study evaluates if combining compartment‐based reconstruction techniques with acquisition‐weighted (AW) Triple Repetition Time Saturation Transfer (TRiST) acquisitions could overcome these challenges.

**Methods:**

Healthy volunteers were scanned with a fully weighted (FW) TRiST protocol twice, and once with an AW TRiST protocol on a 3T MRI. The resulting spectra were reconstructed with conventional Fourier Transform (FT), as well as compartment‐based reconstruction techniques: Spectroscopy with Linear Algebra Modeling (SLAM), Spectral Localization by IMaging (SLIM), and an unweighted mean of the FT spectra (ROI‐FT). *k*_f_ values were calculated and compared across reconstruction methods and acquisition types.

**Results:**

The cardiac *k*_f_ values from FW TRiST were 0.21 ± 0.07 s^−1^ (FT), 0.26 ± 0.08 s^−1^ (SLAM), 0.26 ± 0.07 s^−1^ (SLIM), and 0.30 ± 0.10s^−1^ (ROI‐FT). Corresponding values from AW TRiST were 0.27 ± 0.07 s^−1^, 0.25 ± 0.05 s^−1^, 0.25 ± 0.04 s^−1^, and 0.24 ± 0.08 s^−1^, respectively. No significant differences were observed between FW and AW results. A significant decrease in cardiac PCr/ATP ratios was observed for SLAM and SLIM reconstructed data, suggesting decreased signal contamination from skeletal muscle.

**Conclusion:**

Compartment‐based reconstruction techniques minimize the operator subjectivity present in the current FT method of analyzing TRiST experiments, in addition to reducing skeletal muscle contamination. When combined with an AW acquisition, scan times were reduced by 47% without compromising *k*_f_ accuracy. This method provides a more robust and efficient evaluation of in vivo cardiac metabolism.

## 1 Introduction

Adenosine triphosphate (ATP) is the sole substrate used to directly fuel both cardiac muscle contraction and ion pumps. A high rate of ATP hydrolysis is essential to sustain the continuous mechanical work of the heart. The myocardial ATP pool (˜5 μmol/g wet weight [1]) is relatively small, so to maintain ATP concentrations throughout the cardiac cycle, ATP is primarily generated through oxidative phosphorylation and, to a lesser extent, glycolysis. Additionally, there is a limited phosphocreatine (PCr) pool (˜8 μmol/g wet weight [1]), which serves as an immediate energy buffer. PCr hydrolysis is catalyzed by the enzyme creatine kinase (CK) to help sustain myocardial ATP concentrations. Reduced myocardial CK flux indicates perturbed cardiac energy metabolism and is a common feature of cardiac diseases and is associated with poor outcomes in heart failure (HF) patients [2], making the ability to quantify the forward reaction rate of the CK reaction (*k*_f_) a valuable tool for the assessment of the cardiac energetic state.

Phosphorus magnetic resonance spectroscopy (^31^P MRS) is a noninvasive method for measuring the concentrations of phosphorus‐containing metabolites in vivo [3]. In the heart, the primary sources of ^31^P MRS signal arise from ATP and PCr [4], enabling measurements of metabolite concentration ratios and, when combined with saturation transfer (ST) techniques, assessment of the exchange rates between their pools [5]. ST techniques measure this exchange by selectively saturating one resonance [2], typically γ‐ATP, and observing the change in the PCr signal, which reflects the rate of phosphate transfer catalyzed by CK.

Several ST implementations have been developed to improve efficiency and accuracy of exchange kinetic measurements [6, 7, 8]. Early implementations of ST experiments required multiple steady‐state acquisitions and long repetition times to fit comprehensive exchange models [9], but these were very time consuming, making it impractical for in vivo cardiac studies. More recent developments, such as the Four‐Angle Saturation Transfer [6] (FAST) and two repetition time saturation transfer [7] (TwiST) methods, shortened the total acquisition times by optimizing flip angle and repetition time schemes. The Triple Repetition Time Saturation Transfer [8] (TRiST) method has become one of the most widely used approaches for cardiac studies, having been successfully applied in multiple human investigations at 3T and higher field strengths [10, 11]. TRiST offers a robust balance between accuracy and simplicity, allowing for *k*_f_ calculations from only three acquisitions; however, when implemented with spatial encoding, it still requires relatively long acquisition times (typically 38 min [11] for the protocol used in this study), which limits its use in clinical research.

Currently, when conducting the TRiST protocol, the data is typically acquired with three 1D magnetic resonance spectroscopic imaging (MRSI) localization sequences, implemented as a phase‐encoded chemical shift imaging experiment with spatial localization along a single direction [10]. Postreconstruction, the operator manually selects which single voxel best represents the cardiac spectra, with the data from this chosen voxel used to calculate the cardiac CK reaction rate. As 1D‐MRSI provides localization in only one dimension, and because voxel selection is subjective, this approach remains susceptible to partial‐volume effects and contamination from adjacent skeletal muscle signals, which can compromise quantitative accuracy.

A potential solution to this issue is the combined use of compartment‐based reconstruction techniques [12, 13] where a single spectrum is generated for each predefined region of interest (ROI), removing the potential for human error. This technique has recently been applied to cardiac 3D‐MRSI at 3T, where compartment‐based reconstruction helped reduce the sensitivity to cardiac motion and removed the dependence of the measured PCr/ATP ratio on the operator‐selected voxel [14].

A commonly used method to overcome the traditionally long acquisitions of MRSI is the use of acquisition weighting [14] (AW). AW reduces scan time by prioritizing the acquisition of central *k*‐space data over uniform sampling. In addition to reducing acquisition time, AW dampens sidelobes in the point spread function, minimizing partial‐volume effects and reducing signal contamination from adjacent volumes, even at lower spatial resolution [14].

Therefore, this work aims to evaluate a more robust and time‐efficient approach for quantifying the CK exchange rate in the human heart at 3T by combining an AW TRiST protocol with compartment‐based reconstruction techniques. While each of these approaches has been well validated individually, their integration for cardiac exchange rate quantification using ^31^P MRS has not previously been investigated. The proposed combined approach has been designed to overcome three of the main challenges observed in TRiST studies; operator subjectivity in voxel selection, skeletal muscle contamination, and acquisition durations that are often too long to be viable for routine clinical research. The study comprised two parts, first evaluating the impact of compartment‐based reconstruction on the repeatability of *k*_f_ estimations for fully weighted (FW, i.e., uniformly sampled) TRiST datasets, followed by investigating whether an AW TRiST protocol could reduce the total scan time while preserving accuracy.

## 2 Methods

### 2.1 Data Acquisition, Reconstruction, and Analysis Workflow

This section describes the complete workflow used for the TRiST experiments, including data acquisition, reconstruction techniques applied, and data analysis, including a description of how the repeatability across reconstruction techniques was evaluated.

Seven healthy volunteers (4 M/3F, mean age: 32 ± 3) were scanned in a supine position using a FW TRiST protocol on a Siemens Prisma 3 T MRI system (Siemens Healthineers, Erlangen, Germany) equipped with a multilayer ^31^P surface coil [15]. All participants were scanned under an institutionally approved technical development standard operating procedure.

To implement the TRiST protocol, the vendor's 1D chemical shift imaging sequence was modified to include continuous frequency‐selective saturation of the γ‐ATP resonance while awaiting a trigger from the electrocardiogram, as previously described by Clarke et al. [10]. Following detection of the R‐wave, saturation was maintained for a short delay until diastole, where excitation and data acquisition were performed to reduce cardiac motion sensitivity.

**FIGURE 1:**
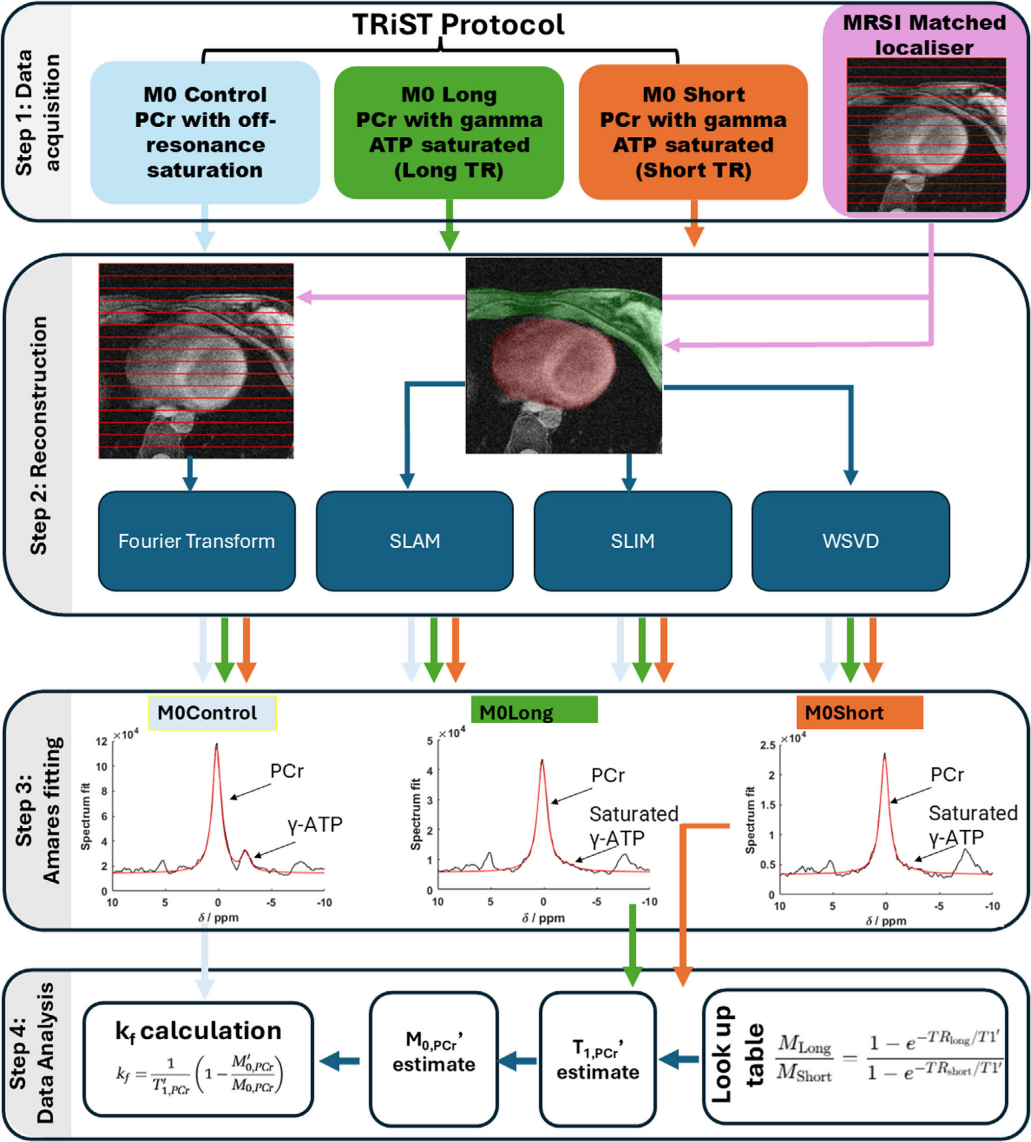
Overview of the workflow used for calculating the forward CK reaction rate using the TRiST protocol.

The TRiST experiment utilized three 1D‐MRSI acquisitions, each with 16 phase‐encoding steps and an FOV of 160 mm along the anterior–posterior direction (the receive sensitivity of the coil defines the borders in the other directions), as illustrated in step 1 of Figure 1:

1. *M0Control*—A control acquisition with a mirrored saturation pulse at a frequency symmetric to γ‐ATP.
2. *M0long*—A long repetition time (TR) acquisition with a DANTE [16] (delay alternating with nutation for tailored excitation) pulse train selectively saturating the γ‐ATP resonance.
3. *M0Short*—A second acquisition with identical γ‐ATP saturation but a short TR.

**FIGURE 2:**
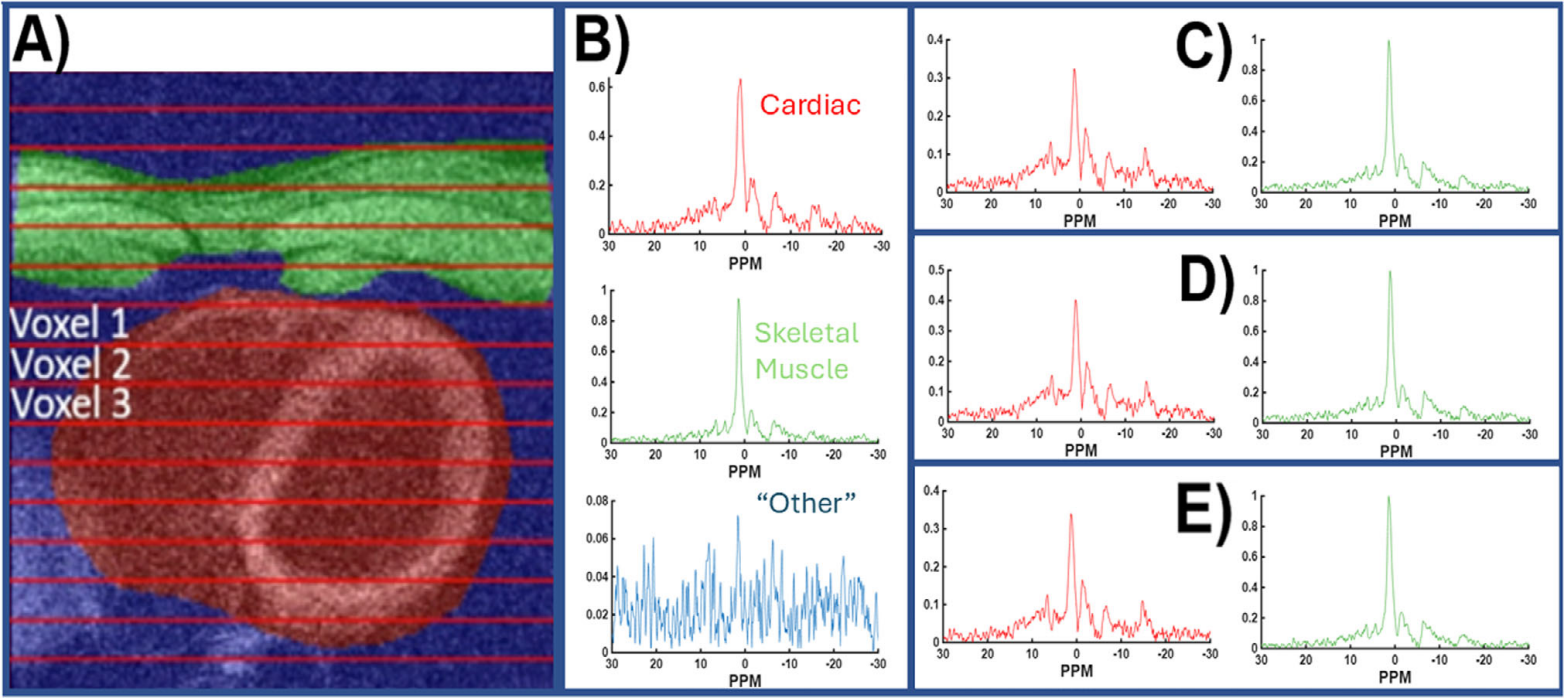
An example of the segmentation mask and the first three cardiac voxels on the localizer (A). The resultant FT reconstruction spectra (B) of the heart (red), skeletal muscle (green), and other (blue) components. Reconstructed cardiac and skeletal muscle spectra obtained using SLAM (C), SLIM (D), and the ROI‐FT (E). For each reconstruction method, spectra are normalized to the maximum signal intensity within that method, therefore *y*‐axis scales differ between reconstruction techniques.

The three MRSI acquisitions had TRs of 15 s, 10 s, and 2 s, with corresponding averages of 2, 8, and 18, respectively. Subjects were instructed to maintain shallow, consistent breathing during the scan. Combined with the long repetition times used in the TRiST protocol, this approach further reduced susceptibility to respiratory‐induced motion artifacts. This was repeated once on each volunteer in separate sessions on the same day. To aid with the analysis process, an MRSI matrix matched gradient echo localizer (Repetition time = 2590 ms, Echo Time = 3.56 ms, Flip angle = 6°, FOV = 160 mm × 160 mm, Matrix size = 192 × 192) was acquired during each session. The cardiac and skeletal muscle compartments were defined with manually drawn ROIs on the localizer. This manual segmentation was performed once per subject and used consistently across all reconstruction methods. To provide consistency in analysis, the ROIs were also used for the Fourier Transform calculations, with the most anterior voxel with > 50% of its volume containing heart selected as the “best” FT spectra. An example of this is provided in Figure 2a.

Raw spectroscopy data was exported from the scanner in the Siemens TWIX data format, allowing each of the reconstruction techniques being investigated to be applied. Data was reconstructed in MATLAB (R2022a, Mathworks, Natick, Massachusetts, USA) using the following reconstruction methods:

Spectroscopy with linear algebra modeling (SLAM) [12] and Spectral localization by imaging (SLIM) [13] are both established compartment‐based MRSI methods that incorporate both acquisition and reconstruction components. In this work, only the reconstruction components were implemented, using the defined ROI marks to define spatial compartments. A detailed mathematical description of these reconstruction methods is provided by Tyler et al. [17].

- *Fourier transform (FT)*: This is the current “gold‐standard,” where a Fourier transformation is applied to the raw data to generate a spectrum for each of the phase‐encoding steps used. The operator must manually select the “best” spectrum to represent the cardiac region.
- *Spectroscopy with Linear Algebra Modeling (SLAM)* [12]: A model‐based reconstruction performed directly in *k*‐space using user‐defined compartment masks to estimate a single spectrum for each region of interest by solving a linear system.
- *Spectral Localization by IMaging (SLIM)* [13]: *A model‐based reconstruction performed in image space, by* modeling the measured signal as the convolution of the true underlying compartment spectra with the system point‐spread function. The resulting linear system is solved to estimate a single spectrum for each ROI.
- *ROI‐averaged Fourier transform (ROI‐FT)*: Following FT reconstruction of the data, the spectra from all cardiac voxels defined by the ROI were averaged by taking the arithmetic mean to generate a single cardiac spectrum. This approach provides a simple, unweighted combination of voxels to reduce operator subjectivity, while maintaining the conventional FT reconstruction workflows. Given the simplicity and ease of integration into existing workflows, ROI‐FT is included as a reference technique against which more advanced compartment‐based approaches can be compared.

As outlined by Step 3 in Figure 1, PCr and ATP amplitudes were extracted by fitting each of the reconstructed spectra using the AMARES algorithm [18] through the MATLAB‐based OXford Spectroscopy Analysis (OXSA) [19] toolbox. By selectively saturating the γ‐ATP signal, the resulting change in PCr signal due to chemical exchange was evaluated to calculate the CK forward exchange rate constant, *k*_f_. The ratio of the PCr amplitudes from M0Short and M0Long was used to estimate T_1,PCr_′ (the effective longitudinal relaxation time of PCr) from a lookup table. This allowed M_0,PCr_′ to be calculated, which was used to estimate *k*_f_ based on the PCr amplitude from M0Control. Monte‐Carlo error propagation was used based on the fitted Cramer‐Rao lower bound. Additionally, PCr/ATP ratios and PCr signal to noise ratios were calculated from the control acquisition. Bland–Altman plots were generated to assess the repeatability in each case, and a paired Wilcoxon signed rank test was used to identify statistically significant differences, with a significance threshold of *α* = 0.05.

### 2.2 Comparison of Acquisition Weighted TRiST Protocol With Fully Weighted

During the same session as the repeat measurement of the FW protocol, an AW TRiST protocol was also conducted. All scan parameters except *k*‐space sampling scheme remained the same. The total scan times for the FW and AW experiments were 38.7 min and 20.3 min, respectively. The same analysis protocol was followed for the AW dataset as the fully weighted experiments.

### 2.3 Phantom Validation Experiment

To investigate whether the compartment‐based reconstruction has the potential to lower contamination from skeletal muscle tissue, a two‐compartment phantom was constructed to mimic distinct cardiac and skeletal muscle spectra. A custom 3D‐printed, dual‐chamber phantom (Figure S1) was designed with adjacent but separate compartments to reproduce the partial‐volume effects encountered in 1D cardiac acquisitions. The “skeletal muscle” chamber contained 100 mM potassium phosphate, while the “cardiac” chamber contained 90 mM potassium phosphate with added sodium hydroxide, resulting in a ˜4 ppm chemical‐shift separation between the two resonances.

Data were acquired using the same 1D‐MRSI sequence and reconstruction pipeline as in the in vivo experiments. The resulting data were reconstructed using FT, SLAM, SLIM, and ROI‐FT methods. Each reconstruction was evaluated for evidence of spectral bleed or signal cross‐contamination between the two compartments.

## 3 Results

### 3.1 Repeatability of CK Reaction Rates With Various Reconstruction Techniques

Figure 2 B‐E displays example spectra generated for the heart and skeletal muscle compartments using FT, SLAM, SLIM, and ROI‐FT reconstruction techniques.

**FIGURE 3:**
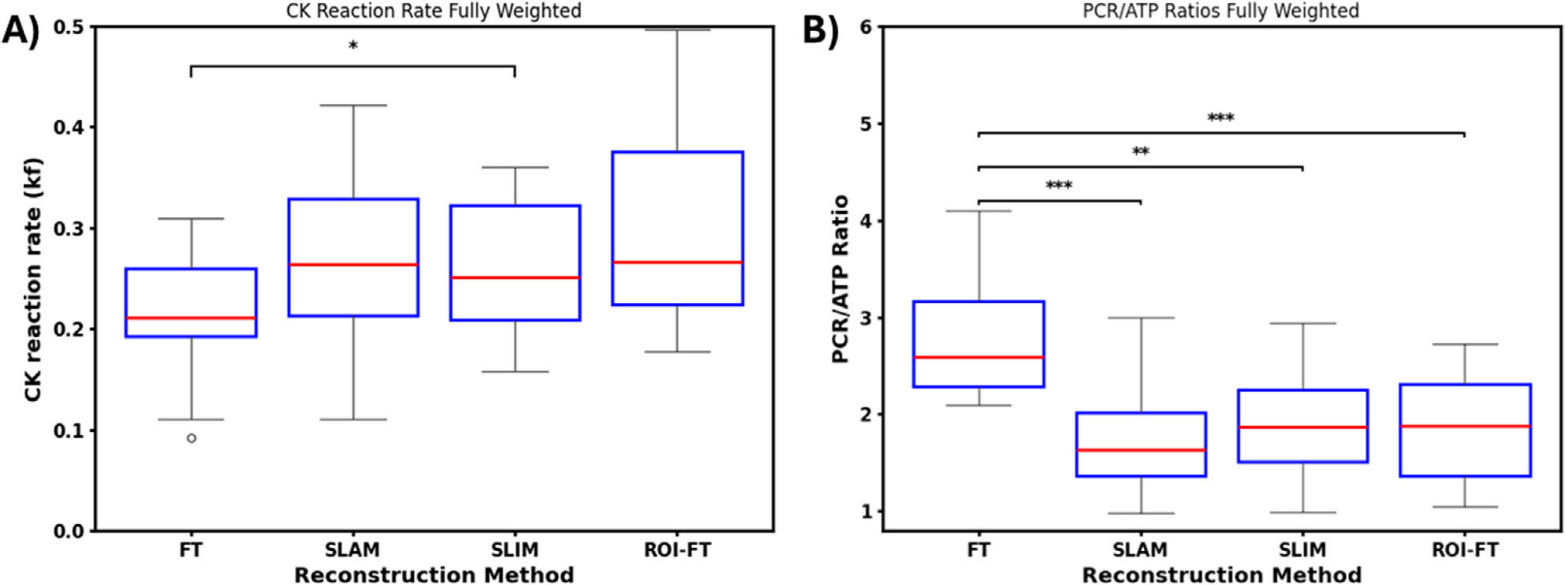
Box plots showing the cardiac CK reaction rates (A) and PCr/ATP ratios (B) for each reconstruction technique. Statistical significance between FT and other methods was assessed using paired Wilcoxon signed‐rank tests. Significance levels are indicated as follows: *P* < 0.05 (*), *p* < 0.01 (**), and *p* < 0.001 (***).

Figure 3A displays the cardiac CK reaction rates calculated from the repeated FW TRiST experiments, using each of the reconstruction techniques. While all methods showed a small increase in *k*_f_ compared with the FT reconstruction, this increase was only statistically significant for the SLIM reconstructed data (*p* < 0.05).

Although the observed variation between FT and the compartment‐based techniques is small, it assumes optimal voxel selection when using FT reconstruction, which is an inherently operator‐dependent process in the current practice. Different voxel selection would lead to increased variability in *k*_f_ measurements, as shown in Figure S2.

Figure 3B displays the cardiac PCr/ATP ratios measured from the reconstructed M0Control spectra. A statistically lower PCr/ATP was observed when any of the compartment‐based reconstruction techniques were used compared to the FT method.

**FIGURE 4:**
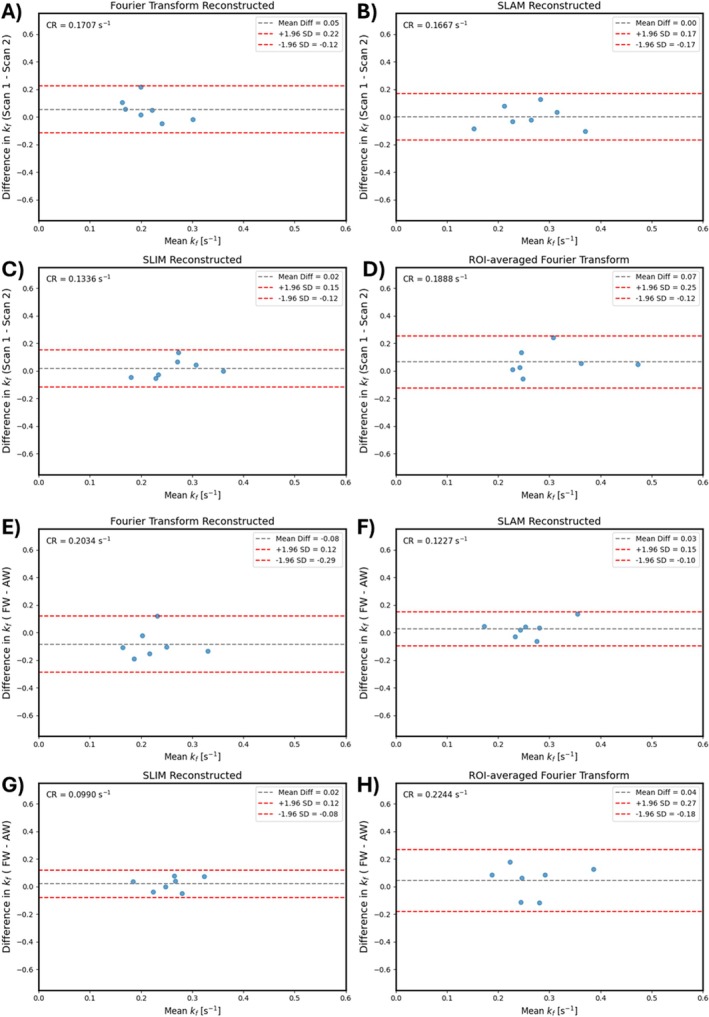
Bland–Altman plots assessing the repeatability of FW measurements of the cardiac CK reaction rate (A–D) and comparing AW and FW measurements (E–H). Results are shown using FT reconstruction (A,E), SLAM reconstruction (B,F), SLIM reconstruction (C,G), and ROI‐FT reconstruction (D,H). The coefficient of repeatability (CR) is shown in the top corner of each plot.

Figure 4A–D presents the Bland–Altman plots comparing cardiac *k*_f_ values obtained from the first and second FW TRiST scans for each reconstruction method. All comparisons displayed minimal bias. Average cardiac *k*_f_ values, PCr/ATP ratios, and PCr SNRs from each reconstruction are summarized in Table 1. The coefficients of repeatability (CR) were 0.17, 0.17, 0.13, and 0.19 s^−1^ for FT, SLAM, SLIM, and ROI‐FT datasets, respectively.

**TABLE 1:** Summary of results showing the average cardiac *k*_f_ values, PCr/ATP ratios, and PCr SNRs.

		Average *k*_f_				Average PCr/ATP ratio				Average PCR SNR			
		FT	SLAM	SLIM	ROI‐FT	FT	SLAM	SLIM	ROI‐FT	FT	SLAM	SLIM	ROI‐FT
Fully weighted	Scan 1	0.24 ± 0.04	0.26 ± 0.08	0.27 ± 0.08	0.33 ± 0.11	3.01 ± 0.80	1.84 ± 0.45	1.87 ± 0.44	2.07 ± 0.46	8 ± 3	10 ± 3	10 ± 3	9 ± 3
	Scan 2	0.19 ± 0.08	0.26 ± 0.08	0.26 ± 0.05	0.27 ± 0.10	2.58 ± 0.45	1.64 ± 0.68	1.86 ± 0.66	1.65 ± 0.57	10 ± 2	8 ± 1	9 ± 2	9 ± 2
Acquisition weighted	Scan 1	0.27 ± 0.07	0.25 ± 0.05	0.25 ± 0.04	0.24 ± 0.08	3.21 ± 1.12	1.65 ± 0.51	1.74 ± 0.47	1.82 ± 0.65	8 ± 2	8 ± 2	8 ± 2	9 ± 2

### 3.2 Comparison of Acquisition Weighted TRiST Protocol With Fully Weighted

To compare the AW and FW protocols, Figure 4E–H displays Bland–Altman plots comparing the *k*_f_ values calculated using AW against the *k*_f_ values from the FW experiment conducted in the same session. Table 1 also includes a summary of average cardiac *k*_f_ values, PCr/ATP ratios, and PCr SNRs for the AW experiments.

The Bland–Altman analysis demonstrated minimal bias and comparable limits of agreement (LOA) across all methods: FT (bias: −0.08 s^−1^, LOA: −0.29 to 0.12 s^−1^), SLAM (0.03 s^−1^, −0.10 to 0.15 s^−1^), SLIM (0.02 s^−1^, −0.08 to 0.12 s^−1^), and ROI‐FT (0.04 s^−1^, −0.18 to 0.27 s^−1^). The corresponding CR was 0.20 s^−1^ for FT, 0.12 s^−1^ for SLAM, 0.10 s^−1^ for SLIM, and 0.22 s^−1^ for ROI‐FT.

### 3.3 Phantom Validation Experiment

Spectra representing the skeletal muscle and cardiac components of the phantom were reconstructed using each of the investigated techniques (Figure S3). The “best” FT voxel still displayed clear contamination from the skeletal muscle component; however, this contamination was largely eliminated using each of the compartment‐based reconstruction methods. Given the minimal residual contamination, the assessment of this dataset was limited to qualitative visual inspection.

## 4 Discussion

The primary aim of this study was to determine whether the application of compartment‐based reconstruction techniques could improve the robustness and repeatability of cardiac CK exchange rate measurements obtained using a TRiST protocol at 3T. While TRiST and all the compartmental reconstruction approaches used have been well established individually, their combination has not been previously investigated in this context. This work therefore, represents the first demonstration of compartment‐based reconstruction applied to TRiST data, allowing for volume‐specific quantification of exchange rates without the need for manual voxel selection. This integration addresses a major limitation of a conventional FT analysis workflow: the operator subjectivity.

The average cardiac *k*_f_ values calculated from the fully weighted TRiST experiments were 0.21 ± 0.07 s^−1^ for FT reconstruction, 0.26 ± 0.08 s^−1^ for SLAM reconstruction, 0.26 ± 0.07 s^−1^ for SLIM reconstruction, and 0.30 ± 0.10 s^−1^ for ROI‐FT. Although the absolute difference was small, SLAM reconstruction and to a greater extent SLIM reconstruction demonstrated improved repeatability, as shown in Figure 4. This was supported by the CRs calculated from the two FW TRiST scans, which were 0.17 s^−1^ for FT, 0.17 s^−1^ for SLAM, 0.13 s^−1^ for SLIM, and 0.10 s^−1^ for ROI‐FT. ROI‐mean FT reconstruction resulted in the highest average calculated *k*_f_ values; however, this also showed increased variability across repeated measurements, while SLAM and SLIM reconstructed data provided more consistent *k*_f_ measurements. By comparing individual spectra produced through each method, it was found that in cases where adequate signal was present throughout the cardiac ROI, the ROI‐FT technique produced results comparable to both SLAM and SLIM reconstructed data; however, in datasets exhibiting rapid spatial signal drop‐off, the ROI‐FT spectra became increasingly influenced by the “best” FT voxel, effectively resulting in the same spectra as with single voxel FT, with additional noise from voxel averaging. These findings indicate that compartment‐based reconstructions can provide more consistent quantification without compromising accuracy.

All calculated *k*_f_ values in this study show good agreement with previous work involving healthy volunteers scanned in the supine position. Clarke et al. [11] reported a mean cardiac *k*_f_ of 0.24 ± 0.12 s^−1^ for supine measurements and noted a systematic increase in *k*_f_ when participants were instead scanned in the prone position, where mean *k*_f_ values were calculated to be 0.32 ± 0.15 s^−1^. This discrepancy has been attributed to respiratory‐ and cardiac‐motion‐induced B_0_ changes in the two body positions. Clarke et al. proposed a linear correction factor to compensate for body position changes which would bring all results in this investigation in line with typical *k*_f_ values reported in other studies using the prone position [20, 21, 22].

The average PCr/ATP ratios calculated from the FW M0Control spectra were 2.80 ± 0.66 for FT reconstruction, 1.74 ± 0.56 for SLAM reconstruction, 1.87 ± 0.54 for SLIM reconstruction, and 1.86 ± 0.54 for ROI‐FT reconstruction. The significantly lower PCr/ATP ratios observed with the compartment‐based techniques likely indicate reduced contamination from skeletal muscle. In contrast, the use of the “best” voxel for FT reconstruction may include signal from the adjacent skeletal muscle, resulting in elevated PCr/ATP ratios. To validate this interpretation, a dedicated two‐compartment phantom experiment was performed (Figure S3). In the phantom, FT reconstruction showed clear spectral bleed from the “skeletal muscle” chamber into the “cardiac” voxels, whereas all other reconstruction techniques largely eliminated this contamination. The phantom experiment provides a controlled “ground truth” test of compartmental separation, independent of acquisition specific factors such as flip angle, repetition time, and saturation efficiency. These results confirm that the lower PCr/ATP ratios measured in vivo reflect improved spatial specificity rather than protocol‐dependent relaxation or flip‐angle effects.

By instead choosing the second most anterior voxel for the cardiac FT analysis, the PCr/ATP measured is lower, 2.30 ± 0.68; however, potential contamination is still observed. Additionally, this would increase the variability in calculated *k*_f_ values. This hypothesis is supported by previous studies reporting myocardial PCr/ATP ratios of approximately 2 in healthy controls [23], compared to values greater than 4 in skeletal muscle [24]. While it is possible to suppress skeletal muscle signal using saturation bands, they were not used in this study due to specific absorption rate (SAR) limitations. The current TRiST protocol was already close to SAR limits, so including additional saturation pulses would have required increased acquisition times or reduced sequence performance.

The second aim of this study was to evaluate whether an AW TRiST protocol could be used to reduce scan times without impacting the accuracy of *k*_f_ measurements. The average *k*_f_ values calculated from the AW TRiST experiments were 0.27 ± 0.07, 0.25 ± 0.05, 0.25 ± 0.04, and 0.24 ± 0.08 s^−1^ for FT, SLAM, SLIM, and ROI‐FT reconstructions, respectively. As demonstrated in Figure 4, no significant differences were observed when compared to the FW TRiST acquisition. The CRs for this comparison were 0.20 s^−1^ for FT, 0.12 s^−1^ for SLAM, 0.10 s^−1^ for SLIM, and 0.22 s^−1^ for ROI‐FT. With the AW protocol being conducted in the same session as one of the FW experiments, this provided confidence that the only difference was the acquisition scheme used. When analyzed using SLAM or SLIM reconstruction, the coefficient of repeatability was further decreased when compared with the FW sessions conducted on separate days. These results confirm that using an AW protocol leads to comparable *k*_f_ values to the FW protocol. The use of AW reduced scan times for the full TRiST protocol from 38.7 min to 20.3 min.

While the results of this study demonstrate strong potential for improving clinical research workflows, several limitations should be considered. First, although compartment‐based reconstructions remove the need for manual voxel selection, ROI definition is still performed manually, introducing some residual operator dependence. Automated segmentation approaches may further improve reproducibility in future work. Second, the use of a 1D‐MRSI acquisition represents a compromise between spatial localization and practical scan times and may be susceptible to partial‐volume effects despite the use of compartment‐based reconstructions. Extension to 2D and 3D MRSI with accelerated encoding strategies may further improve spatial specificity. Finally, all compartment‐based reconstruction techniques assume relatively homogeneous B0 and B1 fields within each compartment, which weren't explicitly corrected for in this work. These inhomogeneities may influence spectral linewidths, signal amplitude, and compartmental signal attribution, potentially contributing to variability in the measured *k*_f_ values. Incorporation of B0/B1 mapping and correction strategies may further improve the quantitative accuracy in future studies.

In summary, compartment‐based reconstruction techniques proved beneficial by reducing operator subjectivity in spectra selection and improving measurement repeatability. While results using SLIM reconstruction were shown to provide the most robust workflow, for 1D‐MRSI acquisitions, the use of an arithmetic mean across relevant voxels also provides an improvement by reducing the operator subjectivity and minimizing signal contamination without requiring significant changes to the existing Fourier transform‐based analysis workflow. For future studies implementing multidimensional MRSI, the ROI‐FT approach may become less viable compared with SLAM or SLIM reconstructions as averaging would not reduce the effects of partial volume contamination or the point spread function. Additionally, the results from the AW TRiST protocol demonstrate that it is a viable alternative to fully weighted acquisitions, providing comparable *k*_f_ measurements while reducing total scan time by approximately 47%. These findings support the integration of both acquisition‐weighting and compartment‐based reconstructions into future protocols aiming to measure cardiac CK flux measurements at 3T.

## Funding

This work was supported by the Sir Henry Dale Fellowship of the Wellcome Trust and the Royal Society (221805/Z/20/Z), by the EU NextGenerationEU through the Recovery and Resilience Plan for Slovakia (09I03‐03‐V02‐00016), British Heart Foundation (FS/SCRF/22/32014, FS/19/18/34252), by the Oxford‐Bristol Myers Squibb Fellowship, and by the Slovak Grant Agencies VEGA (2/0084/26) and APVV (21‐0299).

## Conflicts of Interest

Andrew Tyler has filed a patent in conjunction with Siemens Healthineers in relation to cardiac T1rho imaging. The patent is unrelated to the work in this manuscript, except that a Siemens scanner was used.

## Supporting information


**Figure S1:** Localizer of the custom phantom used to investigate the amount of signal contamination when using different reconstruction techniques (Left). Four voxels around the boundary between the “skeletal muscle” (Top) and “cardiac” components have been highlighted in yellow, red, blue and green, with the resultant Fourier transformed spectra from each of these voxels displayed (Right).
**Figure S2:** Box plot of the CK reaction rates calculated using each reconstruction method, demonstrating the increased variability in results when the second or third “best” voxel is chosen for FT.
**Figure S3:** Localizer of the custom phantom, with ROIs for the skeletal muscle and cardiac components displayed. These ROIs were used to generate a single spectra for each component, for each reconstruction technique.

## Data Availability

The data that support the findings of this study are available from the corresponding author upon reasonable request.
