# Supplementary material for: Faster and More Robust CK Reaction Rate Estimation at 3T Using Acquisition‐Weighted 31P Cardiac 1D‐MRSI With Compartment‐Based Reconstruction
Source: Magn Reson Med. 2026 Apr 26;96(3):1066–75. doi: 10.1002/mrm.70408 (PMC13327432; doi:10.1002/mrm.70408)
Supplement: Supplementary file 1 — Figure S1: Localizer of the custom phantom used to investigate the amount of signal contamination when using different reconstruction techniques (Left). Four voxels around the boundary between the “skeletal muscle” (Top) and “cardiac” components have been highlighted in yellow, red, blue and green, with the resultant Fourier transformed spectra from each of these voxels displayed (Right). Figure S2: Box plot of the CK reaction rates calculated using each reconstruction method, demonstrating the increased variability in results when the second or third “best” voxel is chosen for FT. Figure S3: Localizer of the custom phantom, with ROIs for the skeletal muscle and cardiac components displayed. These ROIs were used to generate a single spectra for each component, for each reconstruction technique. [file MRM-96-1066-s001.docx]

**Supplementary Data**


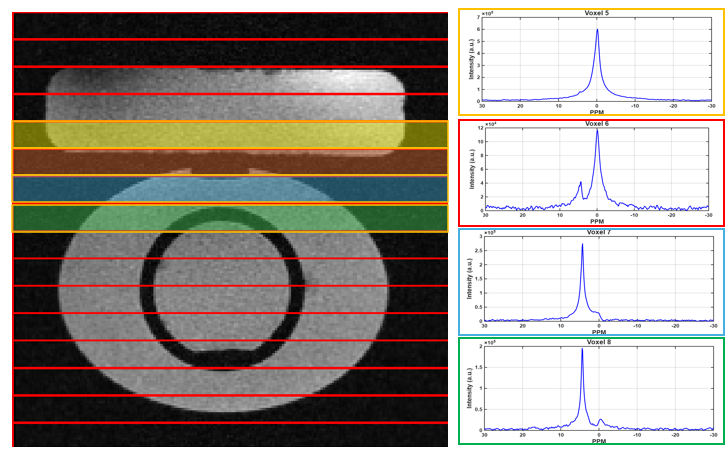


Figure S1: Localiser of the custom phantom used to investigate the amount of signal contamination when using different reconstruction techniques (Left). Four voxels around the boundary between the “skeletal muscle” (Top) and “cardiac” components have been highlighted in yellow, red, blue and green, with the resultant Fourier transformed spectra from each of these voxels displayed (Right)

**
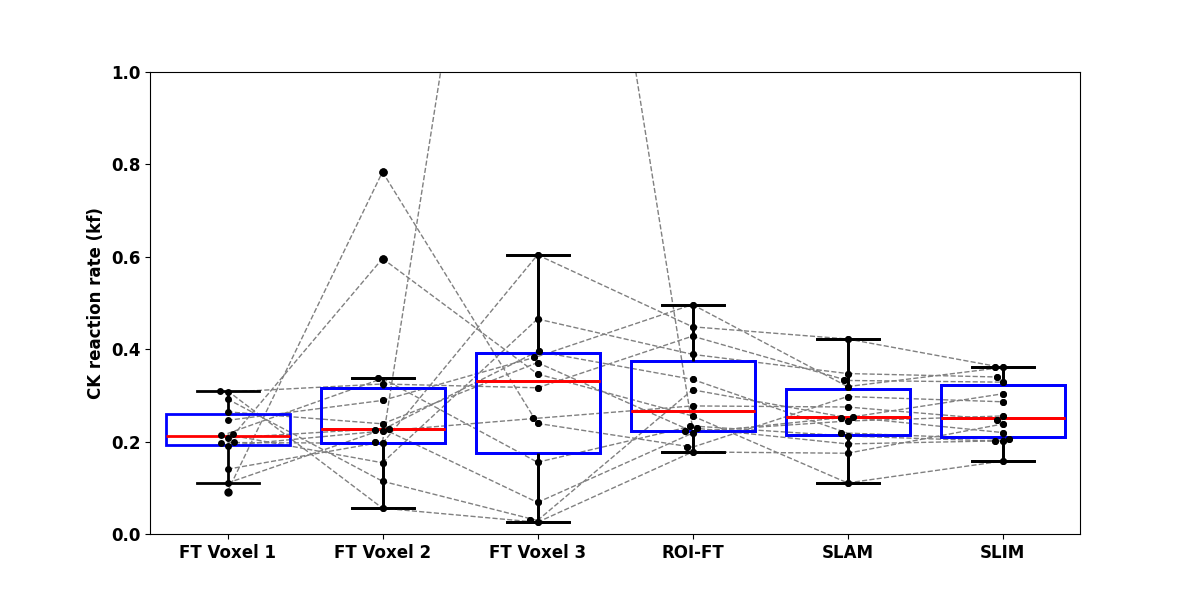
**

Figure S2 Box plot of the CK reaction rates calculated using each reconstruction method, demonstrating the increased variability in results when the second or third “best” voxel is chosen for FT

**
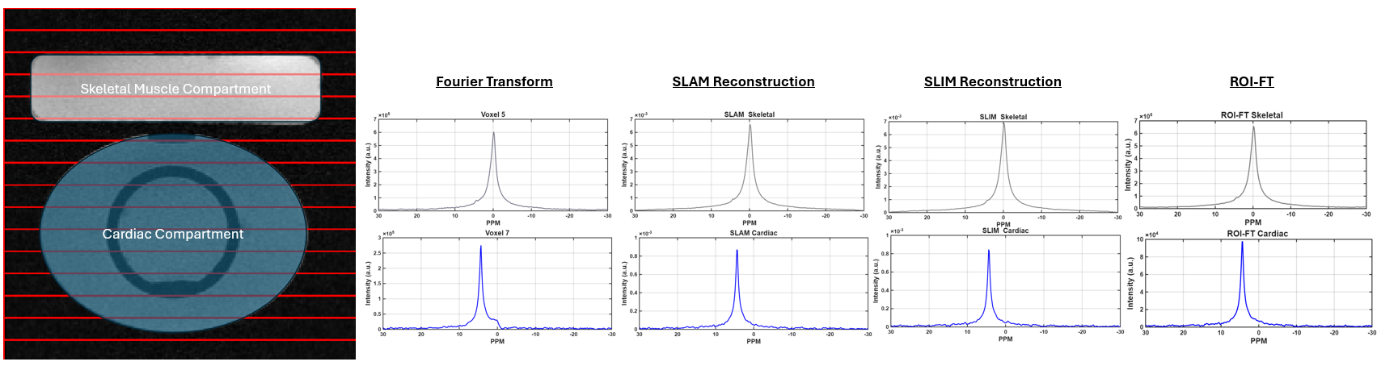
**

Figure S3: Localiser of the custom phantom, with ROIs for the skeletal muscle and cardiac components displayed. These ROIs were used to generate a single spectra for each component, for each reconstruction technique.
